# Use of Vitek 2 Antimicrobial Susceptibility Profile To Identify *mecC* in Methicillin-Resistant Staphylococcus aureus

**DOI:** 10.1128/JCM.00847-13

**Published:** 2013-08

**Authors:** Edward J. P. Cartwright, Gavin K. Paterson, Kathy E. Raven, Ewan M. Harrison, Theodore Gouliouris, Angela Kearns, Bruno Pichon, Giles Edwards, Robert L. Skov, Anders R. Larsen, Mark A. Holmes, Julian Parkhill, Sharon J. Peacock, M. Estée Török

**Affiliations:** Department of Medicine, University of Cambridge, Cambridge, United Kingdoma; Public Health England, Cambridge Microbiology and Public Health Laboratory, Cambridge, United Kingdomb; Cambridge University Hospitals NHS Foundation Trust, Cambridge, United Kingdomc; Department of Veterinary Medicine, University of Cambridge, Cambridge, United Kingdomd; Staphylococcus Reference Unit, Microbiology Services Colindale, Public Health England, Colindale, United Kingdome; Scottish MRSA Reference Laboratory, Stobhill Hospital, Glasgow, United Kingdomf; Department of Microbiology and Infection Control, Statens Serum Institute, Copenhagen, Denmarkg; Wellcome Trust Sanger Institute, Hinxton, United Kingdomh

## Abstract

The emergence of *mecC* methicillin-resistant Staphylococcus aureus (MRSA) poses a diagnostic challenge for clinical microbiology laboratories. Using the Vitek 2 system, we tested a panel of 896 Staphylococcus aureus isolates and found that an oxacillin-sensitive/cefoxitin-resistant profile had a sensitivity of 88.7% and a specificity of 99.5% for the identification of *mecC* MRSA isolates. The presence of the *mecC* gene, determined by bacterial whole-genome sequencing, was used as the gold standard. This profile could provide a zero-cost screening method for identification of *mecC*-positive MRSA strains.

## TEXT

Methicillin resistance in staphylococci is mediated by an altered penicillin-binding protein (PBP2a), which confers resistance to β-lactam antibiotics and is encoded by the *mecA* gene on the mobile element, staphylococcal cassette chromosome *mec* (SCC*mec*) (1, 2). The identification of methicillin-resistant Staphylococcus aureus (MRSA) in diagnostic microbiology laboratories can be achieved by a range of methods, including antimicrobial susceptibility testing, detection of PBP2a by latex agglutination tests, and the molecular detection of the *mecA* gene (3–6).

The description of MRSA isolates from the United Kingdom and Denmark that harbored a divergent *mecA* homologue termed *mecC* (formerly *mecA*_LGA251_) (7) within a novel SCC*mec* XI element was of particular concern because these produced negative results, both by a latex agglutination test and by a PCR assay for *mecA* (8). PCR assays are negative because of divergence in the primer-binding sites, a problem that was rectified by the development of new primers (9–11). Since its original description, *mecC* MRSA has been reported from a number of countries, including France (12), Germany (13, 14), the Netherlands (15), Switzerland (16), the Republic of Ireland (17), Norway (18), Belgium (9), and Sweden (19), and appears to be increasing in prevalence in Denmark (20), highlighting the importance of identifying these isolates. *mecC* MRSA is capable of causing a range of infections and appears to be predominantly community acquired (20). In addition to being found in humans, *mecC* MRSA has also been found in a range of host species (8, 9, 18), with evidence of animal-to-human transmission (21).

Routine diagnostic tests do not, however, provide a mechanism for the identification of *mecC*, which still requires confirmation using PCR assays that are currently available only at reference laboratories (10, 11). The availability of a simple method to identify *mecC* MRSA could allow the monitoring of changes in its distribution and prevalence over time. We made an anecdotal observation, based initially on a small number of strains, that *mecC*-positive MRSA isolates were susceptible to oxacillin but resistant to cefoxitin when tested using the Staph AST-P620 card on the Vitek 2 automated antimicrobial susceptibility testing system (bioMérieux, Marcy l'Étoile, France). This profile differed from the oxacillin-resistant/cefoxitin-resistant profile that is usually observed with *mecA*-positive MRSA isolates.

To test this observation, we assessed the Vitek 2 susceptibility profile and *mec* gene status of a collection of 896 S. aureus isolates which were sequenced using the Illumina HiSeq platform at the Wellcome Trust Sanger Institute (Table 1). Genome sequencing was used as the gold standard for determination of *mec* gene status. Clinical S. aureus isolates were collected as part of routine care and processed at the Cambridge Microbiology and Public Health Laboratory between 2006 and 2012. The isolates included in this study comprised MRSA screening and clinical isolates; 455 were MRSA (*mecA* positive), and 379 were methicillin-susceptible S. aureus (MSSA) (*mecA*/*mecC* negative). We also included 62 *mecC*-positive MRSA isolates, five of which were collected in Cambridge and 57 of which were originally described by García-Álvarez et al. (8).

**Table 1:** Results of Vitek 2 antimicrobial susceptibility testing of Staphylococcus aureus isolates

Identity of S. aureus isolate^*a*^	Total no. of isolates	No. of susceptible and/or resistant isolates/total no. of isolates (%) by Vitek 2^*b*^			
		Oxacillin S and cefoxitin R (S/R)	Oxacillin R and cefoxitin R (R/R)	Oxacillin R and cefoxitin S (R/S)	Oxacillin S and cefoxitin S (S/S)
MRSA *mecC* positive	62	55/62 (88.7)	7/62 (11.3)	0/62 (0)	0/62 (0)
MRSA *mecA* positive	455	4/455 (0.9)	446/455 (98.0)	5/455 (1.1)	0/454 (0)
MSSA *mecA* and *mecC* negative	379	0/379 (0)	0/379 (0)	4/379 (1.1)	375/379 (98.9)

a As determined by bacterial whole-genome sequencing.

b S, susceptible; R, resistant; MRSA, methicillin-resistant Staphylococcus aureus; MSSA, methicillin-susceptible Staphylococcus aureus.

We found that of the 455 *mecA* MRSA isolates, 98.0% were resistant to both oxacillin and cefoxitin (R/R), 1.1% were resistant to oxacillin but susceptible to cefoxitin (R/S), and 0.9% were susceptible to oxacillin but resistant to cefoxitin (S/R) (Table 1). None of the *mecA* MRSA isolates were susceptible to both oxacillin and cefoxitin. Of the 62 *mecC* MRSA isolates, 88.7% were susceptible to oxacillin but resistant to cefoxitin (S/R), 11.3% were resistant to both oxacillin and cefoxitin (R/R), and none were susceptible to both antimicrobials. Of the 379 *mecA*/*mecC*-negative MSSA isolates, 1.1% were resistant to oxacillin but not to cefoxitin (R/S), none were susceptible to oxacillin and resistant to cefoxitin (S/R), and 98.8% were susceptible to both antimicrobials (S/S).

These results generate a sensitivity of 88.7% and a specificity of 99.5% for the identification of *mecC* MRSA based on the S/R profile in a population of both MRSA and MSSA (Table 2). Furthermore, the specificity and sensitivity of identification of *mecA*/*mecC*-negative MSSA, as determined on the basis of susceptibility to both oxacillin and cefoxitin (S/S), are 98.9% (4 false positives of 379 MSSA tested) and 100% (no false negatives), respectively. A recent publication from the United Kingdom Staphylococcal Reference Laboratory estimated the human *mecC* MRSA prevalence rate, as a proportion of phenotypic MRSA, to be 0.5% (5/995) (15). At this prevalence rate, the probability that an oxacillin-susceptible/cefoxitin-resistant profile represents a *mecC* MRSA is 47% (the positive predictive value) and the probability of a non-S/R MRSA not being *mecC* is 99.9% (the negative predictive value). The low prevalence of *mecC* would mean that about half the S/R results would represent *mecA* MRSA. If confirmation of the *mecC* status was required, only a relatively small number of isolates would require further testing by a combined *mecA*/*mecC* PCR assay. The high negative predictive value would enable the correct identification of the vast majority of *mecA* MRSA isolates. The perfect specificity of the oxacillin-susceptible/cefoxitin-susceptible profile as a test for MSSA status ensures that no MRSA (*mecA* or *mecC*) would be wrongly identified as MSSA. The effect of the prevalence rate on the interpretation of tests that do not have perfect sensitivity and specificity highlights the need for data from a formal prevalence survey of *mecC* MRSA. The atypical S/R profile of *mecC* MRSA isolates is likely to be explained by the findings of Kim et al. showing that the *mecC*-encoded PBP2a has a higher relative affinity for oxacillin than for cefoxitin, therefore resulting in higher levels of resistance to cefoxitin than oxacillin (22).

**Table 2:** Diagnostic performance of Vitek 2 antimicrobial profiling to identify *mecC* MRSA^*a*^

Parameter	Value(s)	
	Oxacillin S/cefoxitin R	95% CI
No. of true-positive isolates	55	N/A
No. of false-negative isolates	7	N/A
No. of true-negative isolates	830	N/A
No. of false-positive isolates	4	N/A
Sensitivity (%)	88.7	77.5–95.0
Specificity (%)	99.5	98.7–99.8
Likelihood ratio (positive)	185	69.3–594
Likelihood ratio (negative)	0.11	0.06–0.23

a MRSA, methicillin-resistant Staphylococcus aureus; S, susceptible; R, resistant; 95% CI, 95% confidence interval; N/A, not applicable. The sensitivity is the proportion of true positives testing positive, and the specificity is the proportion of true negatives testing negative. True positives were defined as isolates possessing a *mecC* gene as determined by bacterial whole-genome sequencing. All other isolates were defined as true negatives.

Our findings suggest that in diagnostic laboratories where antimicrobial susceptibility testing is routinely performed using the Vitek 2 system, this method could provide a zero-cost screening method for identification of *mecC*-positive MRSA strains and could potentially be used to monitor changes in the prevalence of *mecC*-positive MRSA over time. It does, however, require examination of the uncorrected Vitek 2 susceptibility results, since the instrument is programmed to override the raw data and report an oxacillin/cefoxitin S/R profile as R*/R, with an explanatory comment to indicate why this has occurred. This highlights one of the limitations of the “expert rules,” which result in automatic amendment of antimicrobial susceptibility data, and the need to educate technologists to examine the uncorrected data to identify possible *mecC* MRSA isolates for confirmatory testing. Further studies to determine whether our findings can be reproduced using other phenotypic antimicrobial susceptibility methods are in progress.
